# Selective Serotonin Reuptake Inhibitor Pharmacokinetics During Pregnancy: Clinical and Research Implications

**DOI:** 10.3389/fphar.2022.833217

**Published:** 2022-02-25

**Authors:** Ethan A. Poweleit, Margaret A. Cinibulk, Sarah A. Novotny, Melissa Wagner-Schuman, Laura B. Ramsey, Jeffrey R. Strawn

**Affiliations:** ^1^ Division of Biomedical Informatics, Cincinnati Children’s Hospital Medical Center, Cincinnati, OH, United States; ^2^ Department of Biomedical Informatics, University of Cincinnati College of Medicine, Cincinnati, OH, United States; ^3^ Department of Pediatrics, Division of Clinical Pharmacology, Cincinnati Children’s Hospital Medical Center, University of Cincinnati College of Medicine, Cincinnati, OH, United States; ^4^ Department of Pediatrics, Division of Research in Patient Services, Cincinnati Children’s Hospital Medical Center, University of Cincinnati College of Medicine, Cincinnati, OH, United States; ^5^ Department of Psychiatry and Behavioral Sciences, University of Southern California, Los Angeles, CA, United States; ^6^ Department of Obstetrics and Gynecology, Division of Maternal-Fetal Medicine, University of Mississippi, Jackson, MS, United States; ^7^ Department of Psychiatry and Behavioral Neuroscience, University of Cincinnati, Cincinnati, OH, United States; ^8^ Department of Pediatrics, Division of Child and Adolescent Psychiatry, Cincinnati Children’s Hospital Medical Center, Cincinnati, OH, United States

**Keywords:** anxiety, depression, pharmacokinctics, pregnancy, SSRI (selective serotonergic reuptake inhibitors)

## Abstract

Pregnancy and associated physiologic changes affect the pharmacokinetics of many medications, including selective serotonin reuptake inhibitors—the first-line pharmacologic interventions for depressive and anxiety disorders. During pregnancy, SSRIs exhibit extensive pharmacokinetic variability that may influence their tolerability and efficacy. Specifically, compared to non-pregnant women, the activity of cytochrome P450 (CYP) enzymes that metabolize SSRIs drastically changes (e.g., decreased CYP2C19 activity and increased CYP2D6 activity). This perspective examines the impact of pharmacokinetic genes—related to CYP activity on SSRI pharmacokinetics during pregnancy. Through a simulation-based approach, plasma concentrations for SSRIs metabolized primarily by CYP2C19 (e.g., escitalopram) and CYP2D6 (e.g., fluoxetine) are examined and the implications for dosing and future research are discussed.

## Introduction

Selective Serotonin Reuptake Inhibitors (SSRIs) are commonly used to treat depression and anxiety across the lifespan, including during pregnancy (Mesches et al., 2020). Among these SSRIs, citalopram, escitalopram, sertraline, fluvoxamine and fluoxetine are most commonly used during pregnancy, while paroxetine is used less frequently secondary to concerns related to the risk of congenital malformations including cardiac malformations (Bérard et al., 2016). In general, when SSRIs are used in pregnancy, there is a consideration of their benefits and risks, including the transient syndrome of neonatal SSRI withdrawal (Moses-Kolko et al., 2005), longer term developmental outcomes of fluoxetine-exposed children and reassuring data suggesting that *in utero* SSRI exposure does not affect IQ and language development (Nulman et al., 1997). Importantly, during pregnancy, plasma SSRI concentrations vary considerably—in part because of a surfeit of pregnancy-related changes in cytochrome P450 (CYP) activity. This variation in SSRI exposure may alter efficacy and tolerability, and necessitate dose adjustment in pregnant people.

Physiologic changes during pregnancy substantially alter SSRI pharmacokinetics Table 1. Pregnancy is associated with delayed gastric emptying, increased gastric pH, increased cardiac output, increased total body water and extracellular fluid space, increased fat compartment, increased renal blood flow and glomerular filtration rate (GFR), decreased plasma albumin concentration, and altered cytochrome P450 activity (Pariente et al., 2016). Further, enhanced elimination and associated decreases in drug exposure (lower peak/trough plasma concentrations) decrease the availability of some medications during pregnancy (Pariente et al., 2016). Yet, despite pregnancy related variation in concentrations of multiple medications—including SSRIs—guidance on SSRI dosing during pregnancy is scarce, with only recommendations from the American College of Obstetrics and Gynecology that a single medication at a higher dose be used rather than multiple medications in treating depression during pregnancy (Tseng et al., 2015; Arnold and Flint, 2017). Further confounding drug metabolism in pregnancy is the potential metabolic contribution of the fetus and placenta. While predominately located in the liver, CYP enzymes are present in a variety of tissues including the human placenta. The fetal liver itself has potential to contribute to maternal drug metabolism, however a significant contribution is unlikely due to the relatively small mass (Hakkola et al., 1998).

**TABLE 1:** Selective serotonin reuptake inhibitors (SSRIs) and cytochrome P450 enzymes responsible for their metabolism, as well as changes in the activity of these cytochromes during pregnancy.

SSRI	Relative change in concentration	References	Enzymes	Activity in pregnancy
Citalopram	↓	Heikkinen et al. (2002)	CYP2C19	Decrease
	↓	Sit et al. (2008)	CYP2D6	Increase
	↓	Westin et al. (2017)	CYP3A4	Increase
Escitalopram	↓	Sit et al. (2008)	CYP2C19	Decrease
	↔	Westin et al. (2017)	CYP2D6	Increase
			CYP3A4	Increase
Paroxetine	↕	Ververs et al. (2009)	CYP2D6	Increase
	↓	Westin et al. (2017)	CYP3A4	Increase
Fluvoxamine	↓	Westin et al. (2017)	CYP2D6	Increase
			CYP1A2	Decrease
Fluoxetine	↓	Heikkinen et al. (2003)	CYP2D6	Increase
	↓	Sit et al., 2010	CYP2C9	Increase
	↔	Westin et al. (2017)		
Sertraline	↓	Sit et al. (2008)	CYP2C19	Decrease
	↓	Freeman et al. (2008)	CYP2B6	Increase
	↑	Westin et al. (2017)	CYP2C9	Increase
	↓	Heinonen et al. (2021)	CYP2D6	Increase

Abbreviations: SSRI, selective serotonin reuptake inhibitor.

↓, decrease in concentration. ↑, increase in concentration. ↕, dependent on the CYP2D6 metabolizer phenotype. ↔, no significant change across pregnancy.

Herein, we will focus on pregnancy-related changes in SSRI pharmacokinetics and how variation is influenced by maternal CYP2C19 and CYP2D6, two cytochrome enzymes whose activity is not only affected by pregnancy, but also affected by genetic variation in the genes that encode these enzymes. Additionally, we will briefly review variation in SSRI exposure during pregnancy using pharmacokinetic modeling simulations and propose next steps in understanding how variation in SSRI pharmacokinetics potentially affect clinical management of pregnant patients in terms of relapse, tolerability and withdrawal symptoms.

## Variation in SSRI Pharmacokinetics

In contemporary clinical practice, treatment guidelines for anxiety or depressive disorders rarely incorporate factors that influence antidepressant exposure (other than dose). Moreover, intrinsic factors that affect SSRI concentrations are rarely considered in clinical trials of SSRIs. As such, the current approach to dosing SSRIs is to typically initiate antidepressant therapy at a ‘starting dose’ and to titrate based on response and tolerability. However, variation in SSRI exposure contributes to differences in efficacy and tolerability (Sakolsky et al., 2011; Strawn et al., 2020). Understanding this variation in pregnancy has important implications given the prevalence of drug discontinuation due to non-response and the burden of depressive and anxiety disorders during pregnancy.

SSRI exposure is affected by many factors (*e.g.*, age, concomitant medications, and CYP activity), as well as medication dose, amount, and dosing frequency. Further, CYP activity is influenced by genetic polymorphisms affecting the amount and/or function of the protein, age-related changes in the maturation of the enzyme and altered enzyme activity due to specific diseases, as well as inflammation. For some SSRIs, CYP activity—which varies among pregnancy—substantially impacts exposure (Area Under the Curve, AUC), maximum concentrations (*C*_*MAX*_), and half-life (t_½_). Pharmacogenetic factors that influence CYP activity are rarely included in current pharmacokinetic models yet understanding these contributions could enhance understanding of differences in SSRI pharmacokinetics, particularly during pregnancy, which itself accentuates this variation in exposure. Such interactions of pharmacogenetics as well as auto- or drug-based enzyme inhibition/induction, must be considered to develop precision dosing algorithms, especially during pregnancy.

## SSRI Pharmacokinetics and Pharmacogenetics

Relationships between pharmacokinetically-relevant genes (e.g., *CYP2D6* and *CYP2C19*) and SSRI exposure have been established over the past 2 decades. Recently, a meta-analysis of 94 unique studies, revealed significant relationships between CYP2D6 and CYP2C19 metabolizer status and escitalopram, fluvoxamine, fluoxetine, paroxetine and sertraline exposure and reciprocal apparent total drug clearance (Milosavljević et al., 2021). Further, in non-pregnant patients, modeling studies and guidelines from the Clinical Pharmacogenetics Information Consortium (CPIC) and The Dutch Pharmacogenetics Working Group recommend that dosing for some SSRIs should consider variation in CYP2D6 and CYP2C19 (Hicks et al., 2015; Brouwer et al., 2021). Recommendations from the Food and Drug Administration (FDA) as well as the European Medicine Agency (EMA) are mixed with regard to variation in CYP2D6 and CYP2C19 and SSRIs. For example, the FDA recommends that coadministration of CYP2D6-metabolized medications with paroxetine should be approached with caution (GlaxoSmithKline, 2012), whereas for fluoxetine, the agency recommends, because fluoxetine inhibits CYP2D6 activity, “individuals with normal CYP2D6 metabolic activity resemble a poor metabolizer… [*eo ipso*] coadministration of fluoxetine with other drugs that aremetabolized by CYP2D6 should be approached with caution (Eli Lilly and Company, 2019).” Additionally, the package insert for fluoxetine notes that concentrations of s-fluoxetine are significantly higher in patients who are CYP2D6 poor metabolizers compared to normal metabolizers. However, the package inserts do not contain specific dosing guidance for either paroxetine or fluoxetine (GlaxoSmithKline, 2012; Eli Lilly and Company, 2019). For citalopram, the FDA-approved package insert recommends, based on an AUC increase of 68% in CYP2C19 poor metabolizers that these individuals not be treated with more than 20 mg/day given the risk of QT prolongation (Allergan USA, 2017). This guidance is reiterated in multiple sections of the document, including the dosing, arming and dosage/administration sections of the document. Further, the document also advises patients with CYP2C19 inhibitors not be treated with doses >20 mg/day (Allergan USA, 2017). Finally, the package inserts for escitalopram and sertraline do not provide any guidance regarding the impact of CYP2C19 phenotype on dosing (Forest Pharmaceuticals, 2009). It is important to note that the FDA labels for most medications were approved before pharmacogenetic associations were well established, and inclusion of pharmacogenetic information occurred retroactively. For example, the anti-coagulant clopidogrel, which was approved in 1997 and had a boxed warning added in 2010 warning “diminished antiplatelet effect in patients with two loss-of-function alleles of the CYP2C19 gene” (Bristol-Myers Squibb and Sanofi Pharmaceuticals Partnership, 2021) However, the drug label still does not require pharmacogenetic testing (Roden and Shuldiner, 2010), which could place the manufacturer at legal risk.

### CYP2D6 and CYP2C19 Activity During Pregnancy

Pregnancy alters the activity of CYP2D6 and CYP2C19. Implicated in the metabolism of approximately 25% of all CYP-metabolized medications, CYP2D6 contributes to the metabolism of multiple SSRIs (e.g., fluoxetine, paroxetine, fluvoxamine). Further, genetic polymorphisms in the *CYP2D6* gene produce phenotypic differences: ultrarapid, normal, intermediate, and poor metabolizers (Caudle et al., 2020). However, during pregnancy, CYP2D6 activity across all phenotypes, except poor metabolizers, increases (Wadelius et al., 1997; Tracy et al., 2005; Ryu et al., 2016). CYP2D6 poor metabolizers have no enzymatic activity given the combination of two no function alleles, so increases in activity for patients with this phenotype may be negligible to nonexistent during pregnancy.

CYP2C19 is the primary enzyme involved in the metabolism of escitalopram, citalopram, and sertraline, as well as many other medications (e.g., proton pump inhibitors, clopidogrel). Similar to CYP2D6, polymorphisms in the CYP2C19 gene produce phenotypes of ultrarapid, rapid, normal, intermediate, and poor metabolizers (Caudle et al., 2017). Small studies have reported CYP2C19 activity decreases during pregnancy (McGready et al., 2003). Like CYP2D6 poor metabolizers, we suspect CYP2C19 poor metabolizers to have trivial decreases in activity, if any at all, during pregnancy due to individuals with this phenotype having two CYP2C19 no function alleles. For several medications, this pregnancy-related variation in CYP2D6 and CYP2C19 activity has been associated with increased clearance of metoprolol (Hogstedt et al., 1985), clonidine, anti-retrovirals and glyburide. Moreover, several lines of evidence suggest the need to titrate several medications during pregnancy (Tasnif et al., 2016).

## SSRI Pharmacokinetics During Pregnancy

Fewer than a dozen *in vivo* and modeling studies have examined SSRI pharmacokinetics in pregnant women (Heikkinen et al., 2002; Heikkinen et al., 2003; Freeman et al., 2008; Sit et al., 2008; Ververs et al., 2009; Sit et al., 2011; Westin et al., 2017), in addition to two modeling-based explorations of SSRI pharmacokinetics in pregnant women (Almurjan et al., 2020; Almurjan et al., 2021). To extend these findings and to illustrate how baseline phenotypic variation in CYP enzymes may affect pregnancy-associated changes in SSRI pharmacokinetics, we simulated escitalopram and fluoxetine concentrations at steady state during pregnancy and compared to a non-pregnant state across metabolizer phenotypes. We estimated the pregnancy-associated changes using MwPharm (version 3.82, Mediware, Czech Republic), a pharmacokinetic modeling program that enables users to approximate a patient’s clearance, volume of distribution, exposure, and concentration of individual medications (e.g., escitalopram and fluoxetine + norfluoxetine) based on previously published parameters (Schenker et al., 1988; Søgaard et al., 2005). A one-compartment and two-compartment model were used for escitalopram and fluoxetine + norfluoxetine, respectively. CYP2C19- and CYP2D6-related differences in clearance for escitalopram and fluoxetine + norfluoxetine, respectively, were determined based on previously published studies (Chang et al., 2014; Steere et al., 2015; Magalhães et al., 2020). Model parameters for each medication were entered, in addition to patient characteristics, including age, body size, sex, and medication/dosing history. Considering patient and medication information, the program simulates a time course of medication plasma concentrations for a patient, in addition to their estimated effects. Physiological changes during pregnancy (e.g., total body weight, creatinine clearance) were based on published parameters (Abduljalil et al., 2012) and NHANES data (Fryar et al., 2021); these parameters were reviewed by a board-certified maternal-fetal medicine physician (SAN) and complete model parameters can be found in the supplement (Supplementary Table S1-S3).

**FIGURE 1:**
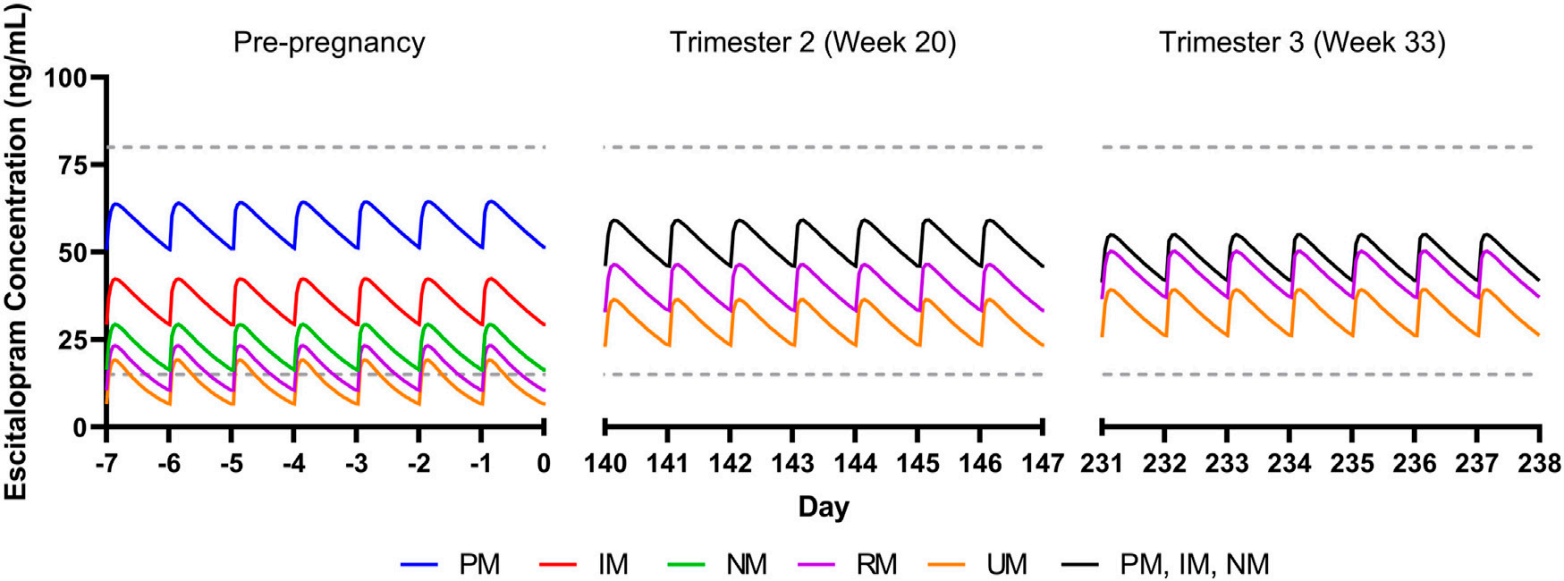
Modeled escitalopram concentrations in pregnancy for CYP2C19 phenotypes. PM, poor metabolizer; IM, intermediate metabolizer, NM, normal metabolizer; RM, rapid metabolizer, UM, ultrarapid metabolizer. Dashed gray lines represent therapeutic trough concentrations (Hiemke et al., 2018).

For a non-pregnant woman treated with escitalopram (20 mg/day), escitalopram concentrations vary significantly across CYP2C19 phenotypes, with rapid and ultrarapid metabolizers having steady state trough concentrations below the lower therapeutic reference range of 15 ng/ml (Figure 1). By trimesters 2 (week 20) and 3 (week 33), there is an estimated decrease in CYP2C19 activity by 62 and 68%, respectively, resulting in trough concentrations for all metabolizer phenotypes within the therapeutic range (McGready et al., 2003; Ke et al., 2014) (Figure 1). CYP2C19 poor, intermediate, and normal metabolizers are expected to have similar escitalopram concentrations by trimester 2 due to activity levels bottoming out, with poor metabolizers having slightly lower concentrations compared to pre-pregnancy due to increases in weight and creatinine clearance (Abduljalil et al., 2012). Escitalopram simulated data are available in the supplement (Supplementary Material).

**FIGURE 2:**
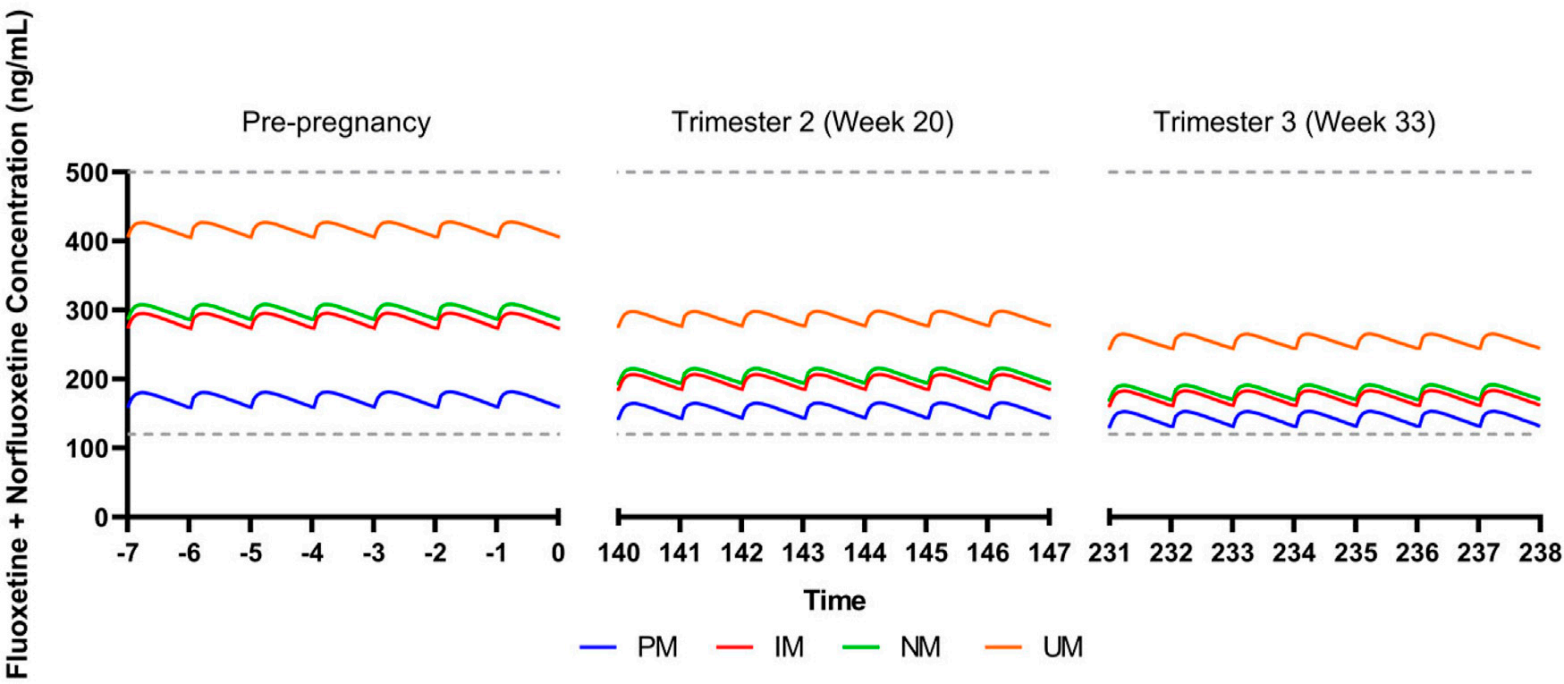
Modeled fluoxetine and norfluoxetine concentrations in pregnancy for patients treated with fluoxetine 40 mg/day. CYP2D6 phenotypes are shown as follows: PM, poor metabolizer; IM, intermediate metabolizer; NM, normal metabolizer; RM, rapid metabolizer; UM, ultrarapid metabolizer. Dashed gray lines represent therapeutic trough concentrations (Hiemke et al., 2018).

We also evaluated the influence of CYP2D6 phenotypes on the pharmacokinetics of fluoxetine and its active metabolite, norfluoxetine. Steady state concentrations were within the expected therapeutic reference range at a dose of 40 mg/day during a non-pregnant state (Hiemke et al., 2018) (Figure 2). By trimester 2 (week 20), CYP2D6 activity is estimated to increase by 131% compared to a non-pregnancy, and trough concentrations of the active moiety for all metabolizer phenotypes are within the therapeutic reference range (Tracy et al., 2005; Abduljalil et al., 2012; Hiemke et al., 2018) (Figure 2). CYP2D6 activity is increased by 137% by trimester 3 (week 33), with trough concentrations still within the therapeutic reference range for all phenotypes (Figure 2). Fluoxetine + norfluoxetine simulated data are available in the supplement (Supplementary Material).

Our simulations reflect differences in escitalopram and fluoxetine pharmacokinetics while accounting for each drug’s primary metabolizing enzyme (CYP2C19 and CYP2D6, respectively), in addition to changes in total body weight and creatinine clearance. While this perspective precludes extensive physiological-based pharmacokinetic modeling that account for additional parameters that are relevant during pregnancy, these simulations reveal significant heterogeneity in SSRI concentrations due to CYP enzymes. Of note, our escitalopram model demonstrates an increase in concentrations for CYP2C19 intermediate, normal, rapid, and ultrarapid metabolizers relative to pre-pregnancy, which contrasts literature showing an overall decrease in escitalopram concentrations throughout gestation (Sit et al., 2008). Whereas we only accounted for CYP2C19, induction of CYP3A4 and CYP2D6 during pregnancy may partially mitigate in CYP2C19 activity, thereby decreasing escitalopram concentrations in later pregnancy (Desta et al., 2002; Tracy et al., 2005). Further, despite trough concentrations being within the therapeutic window for escitalopram and fluoxetine + norfluoxetine, clinicians should monitor changes in target symptoms and tolerability, especially later in pregnancy where SSRI concentrations differ significantly. Models accounting for multiple CYP enzymes involved in the metabolic pathway of these medications, among other pertinent parameters, are needed to further understand the complexity of SSRI pharmacokinetics during pregnancy (Betcher and George, 2020). This may be particularly important in some specific populations and, as an example, in Chinese individuals, CYP2C19 poor metabolizers had a mean 46% increase in fluoxetine C_MAX_ and similar increases in AUC_(0,∞)_ compared to normal metabolizers (Liu et al., 2001). Thus, future investigations of fluoxetine and paroxetine pharmacokinetics, including those in pregnancy, may benefit from including non-CYP2D6 phenotypes. Finally, no studies (or models) have examined the impact of transcription regulators of CYP450 activity in pregnancy, although these transcription regulators (e.g., testis-specific Y-encoded-like protein [TSPYLs]) affect the activity of CYP2C19 and other P450 enzymes (Qin et al., 2018). Recent studies suggest that some single nucleotide polymorphisms may decrease suppression of CYP2C19 expression and boost metabolism of some CYP2C19-metabolized SSRIs, including escitalopram and citalopram, and even alter improvement trajectories in escitalopram and citalopram-treated adults with depressive disorders (Qin et al., 2020).

Beyond these models, two population pharmacokinetic modeling studies previously examined pregnancy-related changes in paroxetine (Almurjan et al., 2020) and sertraline (Almurjan et al., 2021) concentrations with regard to CYP2D6 and CYP2C19 metabolizer status, respectively. These studies aimed to identify “appropriate dose titration strategies to stabilize” medication concentrations within therapeutic ranges during pregnancy. For paroxetine, a significant number of pregnant ultrarapid metabolizers had trough concentrations < 20 ng/ml compared to normal metabolizers and this study suggested that for most phenotypes, pregnant women may require doses >20 mg day to maintain an exposure comparable to 20 mg daily pre-pregnancy (Almurjan et al., 2020). In a virtual modeling study of sertraline pharmacokinetics in pregnancy, trough sertraline concentrations decreased throughout pregnancy. Some of this decreased exposure was related to expansion in maternal volume and decreased albumin. However, titration of sertraline was needed for patients of all CYP2C19 phenotypes. Normal and ultrarapid metabolizers needed doses between 100 and 150 mg daily (throughout the pregnancy). However, poor metabolizers needed a dose of 50 mg daily during the first trimester and then required titration to 100 mg daily during the second and third trimester (Almurjan et al., 2021).

## Therapeutic Drug Monitoring of SSRIs During Pregnancy

Given temporal variation in physiology and drug metabolism throughout pregnancy, therapeutic drug monitoring could facilitate understanding of differences in SSRI exposure and remission during gestation. Though most women take one or more medications during pregnancy, clinical trials often exclude pregnant women, so exposure data are lacking for many medications in pregnant women (NICHD Obstetric and Pediatric Pharmacology and Therapeutics Branch, 2021). Recently, the Eunice Kennedy Shriver National Institute of Child Health and Human Development (NICHD) Obstetric and Pediatric Pharmacology and Therapeutics Branch recognized the knowledge gaps in the use of therapeutics in children, pregnant, and lactating people. The resulting strategic plan that aims to advance safe and effective therapeutics for pregnant and lactating people acknowledges that “a key requirement for the advancement of therapeutics that can restore the foundation for healthy pregnancies is understanding how drug action is altered during normal pregnancy, the post-partum period, and lactation” (NICHD Obstetric and Pediatric Pharmacology and Therapeutics Branch, 2021). Drug action may change during pregnancy because of myriad mechanisms, including pharmacokinetic and pharmacodynamic effects. The NICHD Obstetric and Pediatric Pharmacology and Therapeutics Branch established the Maternal and Pediatric Precision in Therapeutics Hub to aggregate knowledge about maternal and pediatric therapeutics (NICHD Obstetric and Pediatric Pharmacology and Therapeutics Branch, 2021). We look forward to seeing this hub and the research projects funded by this mechanism advance precision therapeutics in pregnancy.

## Conclusion and Future Directions

Pregnancy is associated with induction of many enzymes, including CYP2D6, CYP2C9 (as well as CYP3A4, CYP2E1) and these shifts subtend differences in SSRI metabolism during pregnancy. However, pharmacokinetic data from prospective studies in pregnant women are rare and infrequently consider intrinsic variation in cytochromes activity. Importantly, several approaches may address the dearth of pharmacokinetic data in pregnancy and extend model-based recommendations that have been developed for sertraline and paroxetine (Almurjan et al., 2020; Almurjan et al., 2021). Phlebotomy performed during usual care permits opportunistic sampling, an approach that has been used to examine developmental pharmacokinetics of many medications—including SSRIs—in children (Girdwood et al., 2021). Additionally, population PK studies may provide additional information regarding the pregnancy-related pharmacokinetic changes as they relate to variation in CYP phenotypes. These simulation studies also have the potential to examine the impact of dose changes which may normalize exposure related to pregnancy-related shifts in pharmacokinetic parameters and CYP phenotypes (Almurjan et al., 2020; Almurjan et al., 2021). While understanding the effects of this variation in SSRI pharmacokinetics and the underlying differences in pharmacokinetic genes on SSRI exposure in pregnancy is in its early stages, multiple applications can already be imagined. These include identifying patients at risk of symptomatic worsening as result of decreased SSRI exposure, recognizing SSRI withdrawal symptoms related to increased SSRI metabolism in previously stably treated patients and correctly attributing side effect to pregnancy-related shifts in SSRI exposure. Further, the increasing prevalence of obesity and morbid obesity and effects on adequate medication exposure is poorly understood in pregnancy. Weight may play a significant role in treating depression and anxiety in pregnancy, particularly given that several studies have demonstrated relationships between body mass index and response in antidepressant-treated patients. Incorporating the contribution of obesity on CYP enzyme activity in future models could further enhance our understanding of variation in exposure and thereby decreasing treatment failure. Concomitant medications—which are common in pregnancy—may produce phenoconversion for several CYP enzymes. In pregnant people, the effects of phenoconversion, or even its magnitude throughout pregnancy, are poorly understood. Finally, future studies must examine factors that contribute to pregnancy-related variation in exposure. These factors include changes in renal clearance, which increases during the first trimester, peaks in the second trimester, and diminishes at the end of pregnancy as well as changes in target engagement (e.g., pharmacodynamics).

## Data Availability

The original contributions presented in the study are included in the article/Supplementary Material, further inquiries can be directed to the corresponding author.
