# Supplementary material for: Selective Serotonin Reuptake Inhibitor Pharmacokinetics During Pregnancy: Clinical and Research Implications
Source: Front Pharmacol. 2022 Feb 25;13:833217. doi: 10.3389/fphar.2022.833217 (PMC8916222; doi:10.3389/fphar.2022.833217)
Supplement: Supplementary file 1 [file DataSheet1.docx]

**Supplementary Table 1. Escitalopram Model Parameters**

|  | **Pre-Pregnancy** | | | | | **Trimester 2 (Week 20)** | | | | | **Trimester 3 (Week 33)** | | | | |
| --- | --- | --- | --- | --- | --- | --- | --- | --- | --- | --- | --- | --- | --- | --- | --- |
| **Parameter** | **PM** | **IM** | **NM** | **RM** | **UM** | **PM** | **IM** | **NM** | **RM** | **UM** | **PM** | **IM** | **NM** | **RM** | **UM** |
| **CLm (l/h/70kgBW)** | 13 | 21 | 33 | 45 | 60 | 13 | 13 | 13 | 17.1 | 22.8 | 13 | 13 | 13 | 14.4 | 19.2 |
| **V1 (l/kgLBMc)** | 17.5 | 17.5 | 17.5 | 17.5 | 17.5 | 17.5 | 17.5 | 17.5 | 17.5 | 17.5 | 17.5 | 17.5 | 17.5 | 17.5 | 17.5 |
| **t_1/2_ (h)** | 58.5 | 36.2 | 23.0 | 16.9 | 12.7 | 53.0 | 53.0 | 53.0 | 40.3 | 30.2 | 48.8 | 48.8 | 48.8 | 44.1 | 33.1 |

CLm, central-compartment clearance; V1, central-compartment volume of distribution; t­_1/2_, half life

|  | **Pre-Pregnancy** | | | | **Trimester 2 (Week 20)** | | | | **Trimester 3 (Week 33)** | | | |
| --- | --- | --- | --- | --- | --- | --- | --- | --- | --- | --- | --- | --- |
| **Parameter** | **PM** | **IM** | **NM** | **UM** | **PM** | **IM** | **NM** | **UM** | **PM** | **IM** | **NM** | **UM** |
| **CLm (l/h/70kgBW)** | 28.2 | 28.8 | 40.3 | 50.4 | 28.2 | 37.9 | 53.0 | 66.3 | 28.2 | 39.5 | 55.2 | 69.1 |
| **V1 (l/kgLBMc)** | 31 | 31 | 31 | 31 | 31 | 31 | 31 | 31 | 31 | 31 | 31 | 31 |
| **CL2 (l/h/70kgBW)** | 14 | 14 | 14 | 14 | 14 | 14 | 14 | 14 | 14 | 14 | 14 | 14 |
| **V2 (l/kgLBMc)** | 13 | 13 | 13 | 13 | 13 | 13 | 13 | 13 | 13 | 13 | 13 | 13 |
| **t_1/2_ (h)** | 86.2 | 85.0 | 68.4 | 60.9 | 78.1 | 64.3 | 53.9 | 49.3 | 72.0 | 57.8 | 48.8 | 44.8 |

**Supplementary Table 2. Fluoxetine Model Parameters**

CLm, central-compartment clearance; V1, central-compartment volume of distribution; CL2, peripheral-compartment clearance; V2, peripheral-compartment volume of distribution; t­_1/2_, half life

|  | **Pre-Pregnancy** | | | | **Trimester 2 (Week 20)** | | | | **Trimester 3 (Week 33)** | | | |
| --- | --- | --- | --- | --- | --- | --- | --- | --- | --- | --- | --- | --- |
| **Parameter** | **PM** | **IM** | **NM** | **UM** | **PM** | **IM** | **NM** | **UM** | **PM** | **IM** | **NM** | **UM** |
| **CLm (l/h/70kgBW)** | 20.5 | 8.7 | 7.3 | 4.7 | 20.5 | 11.5 | 9.6 | 6.2 | 20.5 | 12.0 | 10.0 | 6.5 |
| **V1 (l/kgLBMc)** | 15.02 | 15.02 | 15.02 | 15.02 | 15.02 | 15.02 | 15.02 | 15.02 | 15.02 | 15.02 | 15.02 | 15.02 |
| **CL2 (l/h/70kgBW)** | 11 | 11 | 11 | 11 | 11 | 11 | 11 | 11 | 11 | 11 | 11 | 11 |
| **V2 (l/kgLBMc)** | 10 | 10 | 10 | 10 | 10 | 10 | 10 | 10 | 10 | 10 | 10 | 10 |
| **t_1/2_ (h)** | 76.5 | 144.5 | 167.9 | 249.5 | 69.3 | 104.6 | 120.4 | 176.4 | 63.8 | 96.3 | 107.3 | 156.7 |

**Supplementary Table 3. Norfluoxetine Model Parameters**

CLm, central-compartment clearance; V1, central-compartment volume of distribution; CL2, peripheral-compartment clearance; V2, peripheral-compartment volume of distribution; t­_1/2_, half life
